# Supplementary material for: A multi-frequency whole-brain neural mass model with homeostatic feedback inhibition
Source: PLoS Comput Biol. 2026 May 13;22(5):e1013463. doi: 10.1371/journal.pcbi.1013463 (PMC13183287; doi:10.1371/journal.pcbi.1013463)
Supplement: S1 Fig — For the Jansen-Rit model with homeostatic plasticity, the bifurcation diagram (left), associated main oscillation frequency (center), and two representative activity traces with their corresponding power spectral density (PSD; right) are shown for multiple pyramidal firing-rate targets (rows: ρ = 2.5, 2.9, 3.0, 3.2, 3.3 Hz; indicated in each left panel). In the bifurcation diagrams, thick red and black lines denote stable and unstable fixed points, respectively, while green and blue curves indicate maxima and minima of stable (green) and unstable (blue) periodic orbits. The same color convention is used in the center panels to report the oscillation frequency (Hz) along the corresponding periodic branches. In the right panels we show sample time series and PSD, at the labeled input values p. Dots denote bifurcations: black = Hopf bifurcation; purple = saddle-node (either fixed points or limit cycles); blue = Torus or Neimar-Sacker bifurcation; red = branching or pitchfork bifurcation. (PDF) [file pcbi.1013463.s001.pdf]

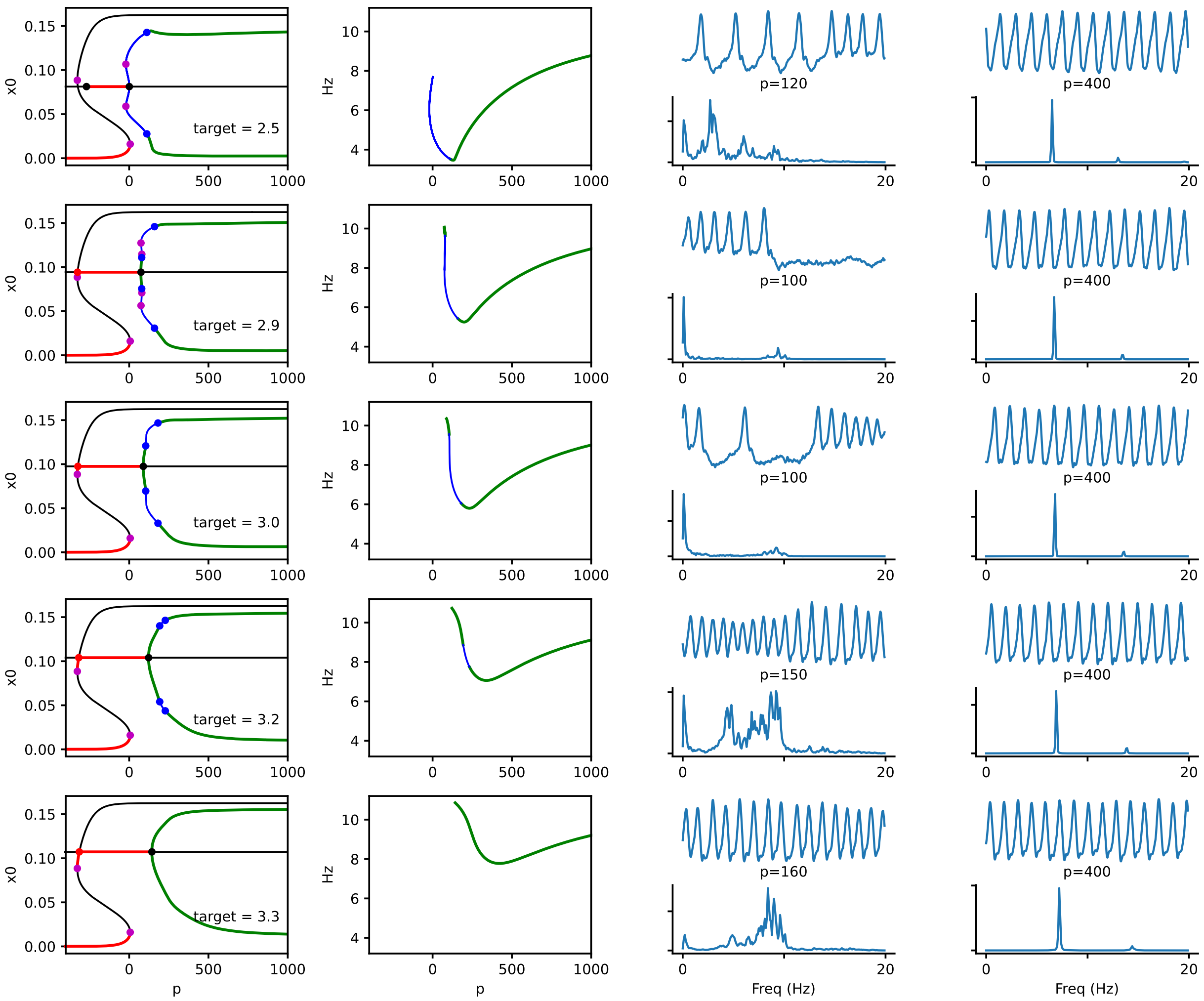

S1 Fig. Extended bifurcation analysis across homeostatic targets. For the Jansen-Rit model with homeostatic plasticity, the bifurcation diagram (left), associated main oscillation frequency (center), and two representative activity traces with their corresponding power spectral density (PSD; right) are shown for multiple pyramidal firing-rate targets (rows: ρ = 2.5, 2.9, 3.0, 3.2, 3.3 Hz; indicated in each left panel). In the bifurcation diagrams, thick red and black lines denote stable and unstable fixed points, respectively, while green and blue curves indicate maxima and minima of stable (green) and unstable (blue) periodic orbits. The same color convention is used in the center panels to report the oscillation frequency (Hz) along the corresponding periodic branches. In the right panels we show sample time series and PSD, at the labeled input values p. Dots denote bifurcations: black = Hopf bifurcation; purple saddle node (either fixed points or limit cycles); blue = Torus or Neimar-Sacker bifurcation; red = branching or pitchfork bifurcation.
