# Supplementary material for: A multi-frequency whole-brain neural mass model with homeostatic feedback inhibition
Source: PLoS Comput Biol. 2026 May 13;22(5):e1013463. doi: 10.1371/journal.pcbi.1013463 (PMC13183287; doi:10.1371/journal.pcbi.1013463)
Supplement: S2 Fig — Left panels show the equilibrium variable x0 (excitatory input to pyramidal neurons) and the extrema of periodic orbits as ρ is varied: thick red and black lines indicate stable and unstable fixed points, respectively, and green/blue curves indicate maxima and minima of stable (green) and unstable (blue) periodic orbits. In the right panels we report the main oscillation frequency (Hz) along the corresponding periodic branches using the same color convention. Dots denote bifurcations: black = Hopf; purple = saddle-node (of fixed points or limit cycles); blue = torus/Neimark-Sacker; red = branching/pitchfork bifurcation. (PDF) [file pcbi.1013463.s002.pdf]

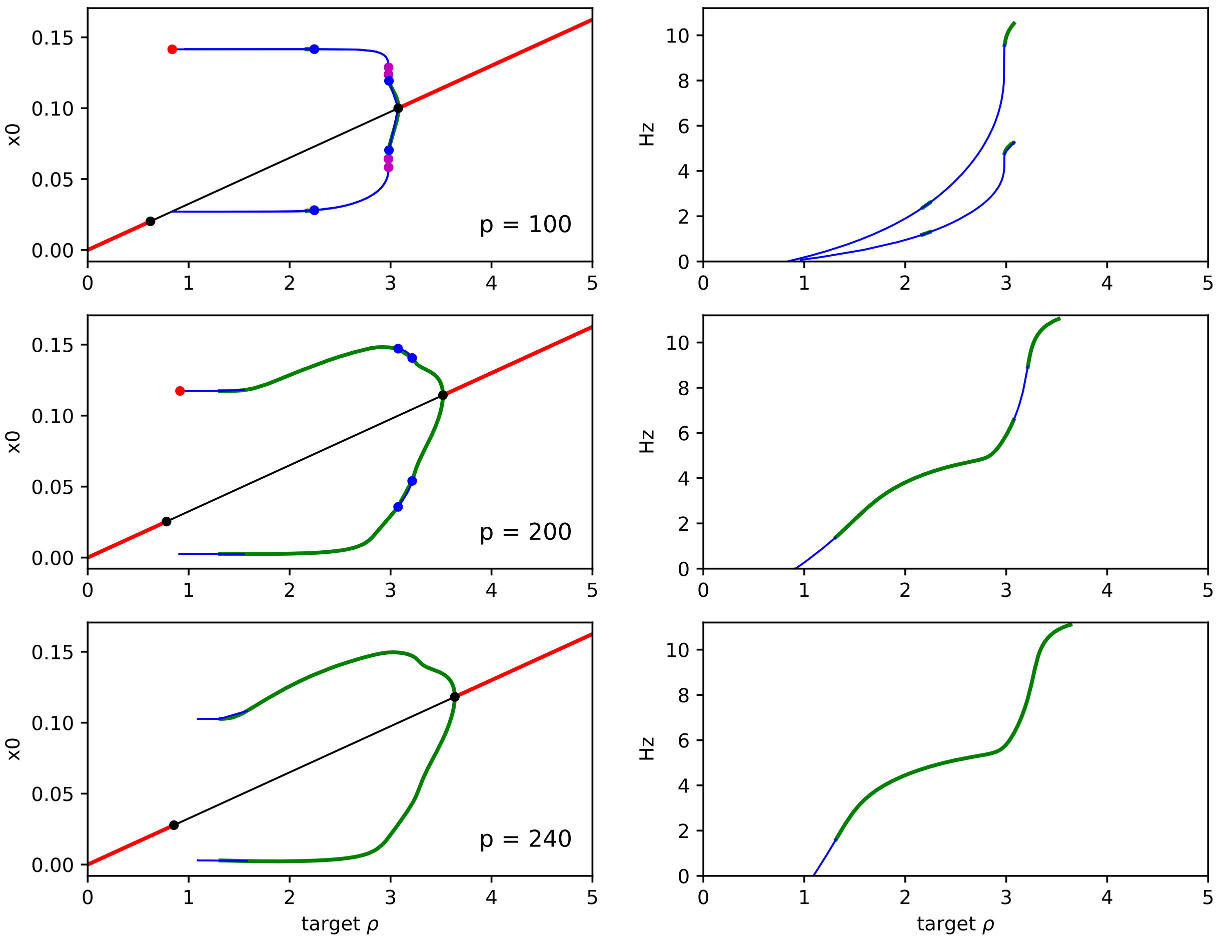

S2 Fig. Bifurcation analysis with respect to the target firing rate ρ at fixed input p. For the Jansen-Rit model with homeostatic plasticity, bifurcation diagrams are shown as a function of the target ρ for three representative constant input values (top to bottom: p = 100, 200, 240). Left panels show the equilibrium variable x_0 (excitatory input to pyramidal neurons) and the extrema of periodic orbits as ρ is varied: thick red and black lines indicate stable and unstable fixed points, respectively, and green/blue curves indicate maxima and minima of stable (green) and unstable (blue) periodic orbits. In the right panels we report the main oscillation frequency (Hz) along the corresponding periodic branches using the same color convention. Dots denote bifurcations: black = Hopf; purple = saddle-node (of fixed points or limit cycles); blue = torus/Neimark-Sacker; red = branching/pitchfork bifurcation.
