# Supplementary material for: A multi-frequency whole-brain neural mass model with homeostatic feedback inhibition
Source: PLoS Comput Biol. 2026 May 13;22(5):e1013463. doi: 10.1371/journal.pcbi.1013463 (PMC13183287; doi:10.1371/journal.pcbi.1013463)
Supplement: S3 Fig — A) The original SC matrix (young connectome). B) Modified SC matrix after reinforcing homotopic inter-hemispheric connections along the anti-diagonal. C) Difference matrix (optimized minus original). (PDF) [file pcbi.1013463.s003.pdf]

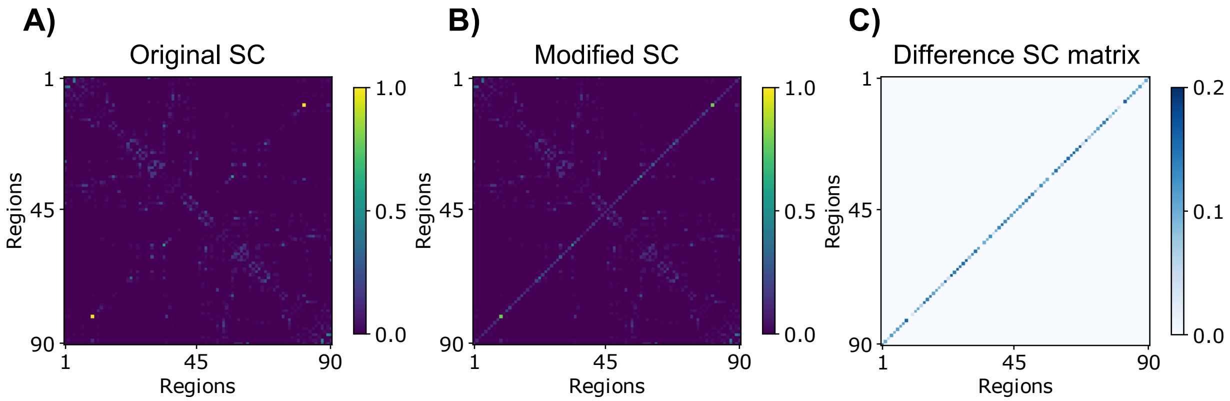

S3 Fig. Structural connectivity (SC) matrices used in whole-brain simulations. **A)** The original SC matrix (young connectome). **B)** Modified SC matrix after reinforcing homotopic inter-hemispheric connections along the anti-diagonal. **C)** Difference matrix (optimized minus original).
