# Supplementary material for: A multi-frequency whole-brain neural mass model with homeostatic feedback inhibition
Source: PLoS Comput Biol. 2026 May 13;22(5):e1013463. doi: 10.1371/journal.pcbi.1013463 (PMC13183287; doi:10.1371/journal.pcbi.1013463)
Supplement: Attachment — Submitted filename: Review_PLOSCompBiology.pdf [file pcbi.1013463.s004.pdf]

This manuscript presents an extended Jansen–Rit whole-brain model that incorporates inhibitory synaptic plasticity (ISP) and multi-frequency cortical subpopulations. The goal is to overcome two limitations of classical neural mass approaches: hyperexcitability and lack of spectral richness. The model is validated across EEG, fMRI, and combined multimodal datasets, including wakefulness and NREM sleep. The work is very interesting; however, some important mechanistic points are unclear, and some claims would benefit from clarification.

Major point:

1- Fig.2: It is not described in a proper way, in the sense that there are many points that are not clear and missing information:
   - In 2A, is there a SNIC bifurcation where the stable limit cycle disappears at the level of the saddle node? If not, what is the mechanism?
   - In 2B pitchfork with three unstable branches (maybe central fixed point is stable?) There is a region in the parameter space in which the dynamics of the system may diverge for low positive external input. For example, the system presents an unstable limit cycle which is not surrounded by any stable attractor. Moreover, it is not clear how the upper unstable fixed point disappears.
   - In 3C there is the same problem about divergence if the system is initiated above the unstable fixed point.

2- Fig.4C: Why is nothing changing for a wide range of the ISP timescale (also, the scale adopted on the figure is not clear)? Since this is the parameter that was added in the present work, I would expect to see its effect, while in this case to me it seems that just by having a constant instead of the differential equation would have been the same. I believe that this point is not well developed in the manuscript since it is fundamental information.

3- The definition and role of the noise in the observed spread in frequency are not mentioned clearly.

Minor points:

1- "…from slower to faster" is unclear. In general, the frequency band should be defined at the first occurrence to make it clear.

2- Improper definition of neural mass models (Wilson-Cowan, Jansen-Rit…) as whole-brain models in the beginning (some whole-brain approaches are network of neural masses)

3- They claim that: “Our previous works have demonstrated that incorporating multiple subpopulations with different oscillatory frequencies within a cortical column can enhance spectral richness [7, 14, 15].”
   Do these models also present the problem of hyperexcitability, or otherwise, which is the main contribution of the current work?
4- In section 2.1 they claim: “The frequency of oscillation is not only dependent on the input p, but also on the target value ρ for the firing rate of the pyramidal population. When ρ is set to 3.5 Hz, oscillations are set very robustly around the α (10 Hz) band”
   This sentence to me is misleading. In the specific case, where just on subpopulation is considered, I would expect ρ, the target firing rate, like the oscillation frequency if the word “target” is expected as something to achieve. If I am misunderstanding, I would suggest either changing the name of the parameter or explaining better what it refers to
5- Fig.3: Not really understanding from the text and the caption what I should see in 3B and 3C (curiosity: why are the frequencies in the gamma and alpha bands appearing in “packages”?). Especially, how should I see that the adjustment of feedback inhibition helps to match the model firing rate?
6- In the discussion, the authors claim: “In our framework, the target firing rate acts as a bifurcation control parameter [10]. By adjusting this parameter, the system can be placed either to the left or right of a Hopf bifurcation.”. In my opinion this is too simplistic as a description of what is happening in the system, considering its high dimensionality and the bifurcation diagram that were presented in which we could observe many kinds of bifurcation apart from the Hopf.
7- In the discussion, they claim: “Our approach aligns with previous computational work modeling sleep-like dynamics through chemical neuromodulation, but here we demonstrate that a single mechanistic parameter (target excitability) is sufficient to reproduce multi-modal empirical data [52-54].”. I believe this sentence is a bit exaggerated because, even if it is true that they show that by changing parameter ρ they can fit experimental data, they are not testing sensitivity about other parameters (it seems to me that also the background input p could be a sensitive parameter).
