# Supplementary material for: A multi-frequency whole-brain neural mass model with homeostatic feedback inhibition
Source: PLoS Comput Biol. 2026 May 13;22(5):e1013463. doi: 10.1371/journal.pcbi.1013463 (PMC13183287; doi:10.1371/journal.pcbi.1013463)
Supplement: Attachment — Submitted filename: Response_R1.docx [file pcbi.1013463.s006.docx]

**RESPONSE TO EDITORS AND REVIEWERS**

**Manuscript ID:** PCOMPBIOL-D-25-01699
**Title:** *A multi-frequency whole-brain neural mass model with homeostatic feedback inhibition*

Dear Drs. van Albada and Berry,

Thank you and the Reviewers for the careful evaluation of our manuscript and for the insightful comments and suggestions. Below, we provide a point-by-point response to all editorial and Reviewer comments. The changes in the manuscript are in red. All modifications in the revised manuscript are tracked in the “Revised Manuscript with Track Changes” file.

We look forward to the evaluation of this new version.

Best wishes,

Agus Ibáñez

**1. EDITOR / JOURNAL REQUIREMENTS**

**Comment E1:**
*Please ensure that the CRediT author contributions listed for every co-author are completed accurately and in full. At this stage, the following Authors/Authors require contributions: Carlos Coronel-Oliveros, Josephine Cruzat, Marilyn Gatica, Raul Gonzalez-Gomez, Hernán Hernandez, Rubén Herzog, Natalia Kowalczyk-Grębska, Fernando Lehue, Vicente Medel, Iván Mindlin, Patricio Orio, Pavel Prado, Enzo Tagliazucchi, and Agustín Ibáñez. Please ensure that the full contributions of each author are acknowledged in the "Add/Edit/Remove Authors" section of our submission form. The list of CRediT author contributions may be found here:*

[*https://journals.plos.org/ploscompbiol/s/authorship#loc-author-contributions*](https://journals.plos.org/ploscompbiol/s/authorship#loc-author-contributions)

**Response E1:**

We have now completed all CRediT roles for every co-author in the submission system and mirrored them in the manuscript.

**Comment E2:**

*Some material included in your submission may be copyrighted. According to PLOS’s copyright policy, authors who use figures or other material (e.g., graphics, clipart, maps) from another author or copyright holder must demonstrate or obtain permission to publish this material under the Creative Commons Attribution 4.0 International (CC BY 4.0) License used by PLOS journals. Please closely review the details of PLOS’s copyright requirements here: PLOS Licenses and Copyright. If you need to request permissions from a copyright holder, you may use PLOS's Copyright Content Permission form.*

*Please respond directly to this email and provide any known details concerning your material's license terms and permissions required for reuse, even if you have not yet obtained copyright permissions or are unsure of your material's copyright compatibility. Once you have responded and addressed all other outstanding technical requirements, you may resubmit your manuscript within Editorial Manager.*

*Potential Copyright Issues:

i) Figure 1A. Please confirm whether you drew the images / clip-art within the figure panels by hand. If you did not draw the images, please provide (a) a link to the source of the images or icons and their license / terms of use; or (b) written permission from the copyright holder to publish the images or icons under our CC BY 4.0 license. Alternatively, you may replace the images with open source alternatives. See these open source resources you may use to replace images / clip-art:

- [https://commons.wikimedia.org](https://commons.wikimedia.org/)
- <https://openclipart.org/>.

ii) Figure 1B appears to have been modified from a previously published figure. Please provide written permission from the copyright holder to publish this under our CC-BY 4.0 license, or remove the figure / replace the image. Please note we do not recommend using standard request forms available on Publishers' websites, as they grant single use rather than republication under an open access license.*

**Response E2:**
We have replaced the next elements in Fig. 1:

Panel A: DTI (source: [File:White Matter Connections Obtained with MRI Tractography.png - Wikimedia Commons](https://commons.wikimedia.org/wiki/File:White_Matter_Connections_Obtained_with_MRI_Tractography.png)) replaced by an image generated by us using FSL ([FSL - FMRIB Software Library](https://fsl.fmrib.ox.ac.uk/fsl/docs/)).

Panel A: All remaining images are original, related to this article, and were generated using Python or Net Brain Viewer for MATLAB.

Panel B: We redesigned this subpanel, and we confirmed that the Figure is original and related to this work. No elements were adapted from previously published figures.

We declared in the “Code and data availability” section the software we used for visualization. All code required to reproduce figures and analyses is now made publicly available.

**Comment E3:**

*In the online submission form, you indicated that Raw data are available on request from Drs. Agustín Ibáñez ([agustin.ibanez@gbhi.org](mailto:agustin.ibanez@gbhi.org)) and Natalia Kowalczyk-Grębska ([nkowalczyk@swps.edu.pl](mailto:nkowalczyk@swps.edu.pl)). All PLOS journals now require all data underlying the findings described in their manuscript to be freely available to other researchers, either

1. In a public repository
2. Within the manuscript itself
3. Uploaded as supplementary information.
   This policy applies to all data except where public deposition would breach compliance with the protocol approved by your research ethics board. If your data cannot be made publicly available for ethical or legal reasons (e.g., public availability would compromise patient privacy), please explain your reasons by return email and your exemption request will be escalated to the editor for approval. Your exemption request will be handled independently and will not hold up the peer review process, but will need to be resolved should your manuscript be accepted for publication. One of the Editorial team will then be in touch if there are any issues.*

**Response E3:**

Thank you for this note. We have uploaded all the empirical data for reproducing our results, including the source EEG time series: [10.5281/zenodo.18841259](https://doi.org/10.5281/zenodo.18841259). In addition, the dataset related to simultaneous EEG-fMRI during sleep transitions is already publicly available (Tagliazucchi et al., 2014, *Neuron*, DOI:10.1016/j.neuron.2014.03.020). The time series (sleep data) in the AAL parcellation were also published in another work of our team at <https://zenodo.org/records/16755776>.

**Comment E4:**

*Please amend your detailed Financial Disclosure statement. This is published with the article. It must therefore be completed in full sentences and contain the exact wording you wish to be published.

1. State the initials, alongside each funding source, of each author to receive each grant. For example: "This work was supported by the National Institutes of Health (####### to AM; ###### to CJ) and the National Science Foundation (###### to AM)."
2. State what role the funders took in the study. If the funders had no role in your study, please state: "The funders had no role in study design, data collection and analysis, decision to publish, or preparation of the manuscript."
3. If any authors received a salary from any of your funders, please state which authors and which funders..
   If you did not receive any funding for this study, please simply state: “The authors received no specific funding for this work.”*

**Response E4:**
We have fixed the funding statement with these recommendations.

**Comment E5:**

*Your current Financial Disclosure states, "Yes ↳ Please add funding details. AI is supported by grants from the Multi-partner consortium to expand dementia research in Latin America [ReDLat, supported by Fogarty International Center (FIC), National Institutes of Health, National Institutes of Aging (R01s AG075775, AG057234, AG082056 and AG083799, CARDS-NIH 75N95022C00031), Alzheimer's Association (SG-20-725707), Rainwater Charitable Foundation - The Bluefield project to cure FTD, and Global Brain Health Institute)], ANID/FONDECYT Regular (1250091, 1210195, 1210176, and 1220995); ANID/PIA/ANILLOS ACT210096; FONDEF ID20I10152, and ANID/FONDAP 15150012. JC is supported by ANID (FONDECYT Postdoctorado #3240042; FONDECYT de Exploración #13240170). This research was supported by the National Science Centre (Poland) Grant: 2013/11/N/HS6/01335, in the years 2013-2017 (to NK-G). The contents of this publication are solely the authors' responsibility and do not represent the official views of these institutions. RH was partially supported by the Ramón y Cajal Fellowship (RYC2022-035106-I) from FSE/Agencia Estatal de Investigación (AEI), Spanish Ministry of Science and Innovation, and the María de Maeztu Program for units of Excellence in R&D, grant CEX2021-001164-M/10.13039/501100011033. ↳ Please select the country of your main research funder (please select carefully as in some cases this is used in fee calculation). UNITED STATES - US".
However, your funding information on the submission form indicates receiving no funds.
Please indicate by return email the full and correct funding information for your study and confirm the order in which funding contributions should appear. Please be sure to indicate whether the funders played any role in the study design, data collection and analysis, decision to publish, or preparation of the manuscript.*

**Response E5:**
Now, the form and the corresponding manuscript section state the same funders.

**Comment E6:**

*Kindly revise your competing statement in the online submission form to align with the journal's style guidelines: 'The authors declare that there are no competing interests.'*

**Response E6:**
We have made these changes.

**2. REVIEWER #1**

**Comment 1.1:**
*The authors propose and evaluate a modification of the Jansen Rit neural mass model combining two extensions : incorporation of two subpopulations at each node providing oscillatory activity on two different frequencies, and a plasticity mechanism to adjust the inhibitory freedback to maintain target firing rates.

In particular, the work presented here builds on previously published models implementing the two individual extensions, evaluates the impact of the plasticity on the bifurcation structure, and demonstrates the capacity of the resulting model to reproduce functional connectivity for both EEG and fMRI in awake and sleep brain states. The manuscript is overall easy to follow and the presented analysis is clear.

If I understand it correctly, both of the extensions have been published before: multiple populations embodying different kinetics in e.g. (1, 2), and the plasticity mechanism introduced in (3). Currently, it is difficult to appreciate what contributions the authors make here before engaging with the manuscript in depth. More precise formulation of the contributions already in the abstract and introduction would help the reader to understand these relationships.*

**Response 1.1:**
Thank you for this comment and positive evaluation of our work. We revised the Abstract, Introduction, and Discussion to make our contributions explicit upfront. “The novelty has been better clarified by integrating the ISP into a multi-frequency JR whole-brain model and shows it (i) expands the oscillatory regime (bifurcation structure), (ii) is necessary for multi-band EEG-FC fitting, and (iii) provides a closed-loop excitability control that reproduces wake/NREM multimodal constraints.

We have made the following changes to the manuscript:

Abstract:

“[...] Here, we developed and characterized a new version of a multi-frequency Jansen-Rit neural mass model aimed at overcoming these limitations. Our model incorporates inhibitory synaptic plasticity (ISP), which adjusts inhibitory feedback onto pyramidal neurons to clamp their firing rates around a target value. [...] Bifurcation analysis showed that ISP increases the parameters’ range in which the model exhibited sustained oscillations [...] The ISP was necessary in the multi-frequency model to successfully fit EEG functional connectivity across frequency bands. Finally, ISP-controlled reductions in excitability reproduced both the slow-wave activity and the reduced connectivity in NREM sleep.”

Introduction:

“Here, we build on a previously developed multi-frequency neural mass model used in dementia [1, 2] based on the Jansen-Rit equations [2, 3] to introduce a biologically motivated inhibitory synaptic plasticity (ISP) mechanism [7, 9, 10].”

“In this work, we characterized a biophysically inspired multi-frequency model [...] which integrates ISP to stabilize network dynamics [4, 5]. [...] Finally, we extend the model to simultaneously fit both EEG power spectra and fMRI FC during wakefulness and NREM sleep…”

Discussion:

“Our model extends the classic Jansen-Rit model by incorporating ISP to modulate excitability and control the oscillatory regime of each subpopulation. In our framework, the target firing rate is a control/input parameter of the closed-loop system (via ISP) that shifts the operating point (mean firing rate / mean inhibitory gain), thereby moving the system through parameter space [4]. [...] The ISP mechanism also provides a practical alternative to heuristic procedures that pre-estimate node-wise feedback inhibition to maintain stable whole-brain dynamics […] In contrast, ISP adjusts inhibition online based on ongoing activity, which avoids repeated recalibration when parameters change and provides direct control of firing rates at the regional level.”

**Comment 1.2:**
*The dependence of the bifurcation structure on the target firing rate presented in Fig. 2B-C is an interesting feature of the model. It would be great to provide the diagrams for more values of target firing rate (e.g. as supplementary) to help to build the intuition on the sensitivity to this parameter.*

**Response 1.2:**

Following the reviewer’s suggestion, we are now including a supplemental Figure (S1 Fig.) that includes the bifurcation diagram of the model at more values of the target ρ. In addition, we inlcuded bifurcations with respect to the ρ parameter at three different input p values (Fig. S3). We attempted to perform 2-parameter bifurcations of some interesting points, but the high dimensionality of the model and the existence of several timescales make the model numerically unstable for that type of continuation analysis.

As shown in the original submission, when target = 2.5 the system undergoes a supercritical Hopf bifurcation at p = 97 that originates an unstable periodic orbit. This orbit then becomes stable at p=111 via a torus bifurcation. Somewhere between 2.7<target<2.8, the supercritical becomes subcritical. However, there remains a p range for which the orbit becomes unstable again via a pair of torus bifurcations. This persists until a point in 3.2<target<3.3, where the periodic orbit remains always stable. Most likely, the remains of this unstable orbit cause the slowing down of the oscillation for 200<p<500, after which the increase in p only causes an increase in oscillation frequency.

“S1 Fig. Extended bifurcation analysis across homeostatic targets. For the Jansen-Rit model with homeostatic plasticity, the bifurcation diagram (left), associated main oscillation frequency (center), and two representative activity traces with their corresponding power spectral density (PSD; right) are shown for multiple pyramidal firing-rate targets (rows: ρ = 2.5, 2.9, 3.0, 3.2, 3.3 Hz; indicated in each left panel). In the bifurcation diagrams, thick red and black lines denote stable and unstable fixed points, respectively, while green and blue curves indicate maxima and minima of stable (green) and unstable (blue) periodic orbits. The same color convention is used in the center panels to report the oscillation frequency (Hz) along the corresponding periodic branches. Right panels show sample time series and PSD, at the labeled input values p. Dots denote bifurcations: black = Hopf bifurcation; purple saddle node (either fixed points or limit cycles); blue = Torus or Neimar-Sacker bifurcation; red=branching or pitchfork bifurcation.”

**“S2 Fig.** Bifurcation analysis with respect to the target firing rate *ρ* at fixed input *p*. For the Jansen-Rit model with homeostatic plasticity, bifurcation diagrams are shown as a function of the target *ρ* for three representative constant input values (top to bottom: *p* = 100, 200, 240). Left panels show the equilibrium variable x0 (excitatory input to pyramidal neurons) and the extrema of periodic orbits as *ρ* is varied: thick red and black lines indicate stable and unstable fixed points, respectively, and green/blue curves indicate maxima and minima of stable (green) and unstable (blue) periodic orbits. Right panels report the main oscillation frequency (Hz) along the corresponding periodic branches using the same color convention. Dots denote bifurcations: black = Hopf; purple = saddle-node (of fixed points or limit cycles); blue = torus/Neimark-Sacker; red = branching/pitchfork bifurcation.”

**Comment 1.3:**
*In addition, the time-scale of plasticity mechanism doesn't seem to play a role (Fig. 4C), which I'd think means that after the initial transient the $C_4$ parameter remains constant. Is this the case? If so, isn't then the plasticity mechanism effectively part of the fitting procedure? In other words, wouldn't it be possible to reach similar results to adjust $C_4$ together with changing coupling strength?*

**Response 1.3:**
We appreciate this point. We clarified in the revised manuscript that ISP adapts the feedback inhibition parameter *C^4^* during an initial transient and then *C_4_* converges to a steady value for a given parameter set (new panel Fig. 4G). Regarding whether ISP is effectively part of the fitting procedure, while it is in principle possible to pre-estimate node-wise *C_4_* values for a given coupling setting, this mapping is not generally portable across parameter changes. We added a new whole-brain analysis showing that the steady-state *C_4_* values scale with nodal strength in a coupling-dependent manner (Fig. 4E) and that this relationship changes systematically with global coupling (Fig. 4F). This illustrates that pre-estimation of *C_4_* would require repeated recalibration across parameter space, whereas ISP provides an online, closed-loop mechanism that automatically adjusts *C_4_* to maintain the target firing rate as parameters (e.g., coupling strength) change.

Results. Section 2.2:

“Across whole-brain simulations, we examined how ISP sets out the spatial distribution of feedback inhibition and how the ISP timescale affects the adaptation dynamics (Fig. 4E-G). For each value of global coupling *K*, the time-averaged feedback inhibition (mean C_4_) was computed at each node and related to the nodal strength of the structural connectivity matrix (Fig. 4E). Mean C_4_ increased with nodal strength, and this relationship was well approximated by a linear regression for each *K* value. Then the slope of this regression was expressed as a function of *K* (Fig. 4F), showing that feedback inhibition changes systematically with global coupling. Importantly, while this mapping could in principle be used to pre-compute a set of node-specific C_4_ values for a given *K*, the regression slope (and thus the inferred C_4_ distribution) also depends on other model parameters (e.g., the α/γ mixture *r*^α^), limiting the generalizability of any fixed pre-computed inhibition across the full parameter space. To assess the role of the ISP time constant, we quantified the convergence time of the plasticity variable in noise-free simulations (Fig. 4G). Convergence timescale (seconds) was defined as the first time at which the ROI-averaged C_4_ reached 63.2% of its total change from the initial value to its final steady-state value. Across the tested time constant values, this parameter primarily modulated the convergence timescale, while the steady-state oscillatory regime (and the normalized PSD, Fig. 4C) remained largely unchanged once the system had converged.”

Results. Fig. 4:

“Fig. 4. Modulation of oscillatory frequency in whole-brain simulations. Normalized power spectral density (PSD) as a function of: A) global coupling, *K*, in the whole-brain model, with a fixed target of *ρ* = 2.5 Hz. B) target (desired) firing rate in the whole-brain model, with a fixed *K* = 0.5. C) inhibitory synaptic plasticity (ISP) time constant, *τ*. The values shown here are the average of 50 random seeds. D) Example of EEG traces for a fixed *K* = 0.5, *r*^α^ = 0.5, *τ* = 2 sec, and different values of *ρ*. E) Relationship between the nodal strength, from the structural connectivity matrix, and the time-averaged feedback inhibition (mean *C_4_*), for different values of global coupling *K*. F) Slopes of the linear regressions in (E), as a function of global coupling. 10 random seeds were used for these simulations. G) Convergence time vs ISP timescale. The convergence time (seconds) was defined as the first time the ROI-averaged *C_4_* reached 63.2% of its total change from the initial to the final steady value in a noise-free simulation.”

Discussion:

“The ISP mechanism also provides a practical alternative to heuristic procedures that pre-estimate node-wise feedback inhibition to maintain stable whole-brain dynamics, as implemented, for example, in the Jansen-Rit [6] and Dynamic Mean Field models [7]. In these approaches, the inferred feedback inhibition is typically tied to a specific choice of coupling and local parameters and therefore becomes sensitive to changes in other model settings, making it impractical to map and fine-tune feedback inhibition across the full parameter space. In contrast, ISP adjusts inhibition online based on ongoing activity, which avoids repeated recalibration when parameters change and provides direct control of firing rates at the regional level. This allows excitability to be set explicitly for individual brain areas through a target firing rate, whereas the mapping between excitability and other model parameters is often indirect and harder to interpret.”

Methods. Section 4.6:

“Using the same whole-brain simulations described above (ISP enabled), we quantified how the spatial distribution of feedback inhibition relates to the structural connectivity topology. For each simulation, we computed the nodal strength of each brain area as the sum of its structural connectivity weights (row-sum of the SC matrix). We then computed the time-averaged feedback inhibition (mean C_4_) for each node by averaging C_4_ over time after 20 sec simulations, discarding the initial 5 sec transient, using 10 random seeds. For each value of the global coupling *K*, we fit a linear regression between nodal strength and mean C_4_ across nodes and store the regression slope (Fig. 4). Unless otherwise stated, these analyses were performed with *r^α^* = 0.5, *ρ* = 2.5 Hz, and *τ* = 2 sec.”

“To quantify how the ISP time constant τ affects adaptation dynamics, we ran noise-free whole-brain simulations (σ = 0) with ISP enabled and fixed parameters *K* = 0.5, *r^α^* = 0.5, and *ρ* = 2.5 Hz, only 1 random seed (noise-free simulation). Simulations had a total duration of 120 sec. We swept *τ* over the values *τ* ∈ [10^-3^, 10^-2^, 10^-1^, 1, 10, 100] sec. For each *τ*, we computed the ROI-averaged C_4_(*t*) and defined a convergence timescale as the first time at which C_4_(*t*) reached 63.2% (1 − 1/e) of its total change from an initial baseline $C_{4}^{0}$ to its final steady value $C_{4}^{\infty}$, where $C_{4}^{0}$ was estimated from the first 2 sec and $C_{4}^{\infty}$ from the last 10 s of the simulation (Fig. 4).”

**Comment 1.4:**
*The discussion of the reproduction of sleep and wake activity is not sufficient in my opinion. The authors state, that their "approach aligns with previous computational work modeling sleep-like dynamics through chemical neuromodulation", however I don't see this alignment especially with respect to the work utilizing the AdEx+adaptation model (refs 52 and 55). There the sleep-like slow-waves are arising from up- and down-state transitions driven by the fluctuation of the adaptation on the slow time-scale, whereas in this work, the sleep-like dynamics are result of the dominance of the slower oscillating population. As the bistable approach reflects the empirical observations better, a more precise discussion would be helpful for the readers to be able to appreciate the differences.*

**Response 1.4:**
Thank you for your observation. We revised the Discussion to clarify that sleep-like dynamics emerge from shifts in excitability and coupling within a limit-cycle regime, relate this to other whole-brain NREM models, and acknowledge the lack of an AdEx/adaptation mechanism as a limitation.

Discussion:

“In whole-brain models where NREM-like slowing is induced by reducing excitability (e.g., decreasing recurrent excitation and/or increasing global coupling), slow-wave-like activity can emerge without explicitly modeling bistable up/down states [8, 9]. We recover the same qualitative mechanism: oscillatory frequency decreases when lowering the target firing rate (ρ) and/or increasing global coupling (K), consistent with our network simulations. [...] this mechanism differs from adaptive exponential neuronal models [10], where NREM slow waves arise from noise-driven transitions between up and down states governed by slow adaptation; our model does not explicitly implement bistability and instead captures sleep-associated slowing via controlled shifts in excitability and coupling within an oscillatory (limit-cycle) regime.

[...]

Second, several mechanisms can modulate E/I balance, beyond ISP, that we did not consider in this work [...] as well as adaptation-driven bistability (up/down state transitions) as implemented in adaptive exponential-based neuronal models [10].”

**Comment 1.5:**

*Some of the methodological choices are stated, but not justified. It is not clear to me, why only 82 regions of the AAL parcellation were used in the EEG simulations, but all 90 regions were used for fMRI simulations. Also, the homotopic connections were strenghtened to compensate underestimation by DTI, however more details would be helpful to understand this step (e.g. a plot of the mask added to the SC).*

**Response 1.5:**
We appreciate your point. Our decision is related to the fact that fMRI FC includes subcortex where EEG source present reliability concerns. Now we clarified that 82 areas from the AAL parcellation (cortical regions plus hippocampus and amygdala) were retained because source-reconstructed EEG is generally less reliable for deep subcortical structures, whereas hippocampal and amygdalar sources can be estimated more robustly than most other subcortical regions in this framework:

<https://www.nature.com/articles/s41467-019-08665-5>

<https://academic.oup.com/braincomms/article/5/1/fcad023/7030489>

We also added a new S3 Fig. showing the structural connectivity matrix and the corresponding SC difference matrix used in this study.

“S3 Fig. Structural connectivity (SC) matrices used in whole-brain simulations. A) The original SC matrix (young connectome). B) Modified SC matrix after reinforcing homotopic inter-hemispheric connections along the anti-diagonal. C) Difference matrix (optimized minus original).”

Methods. Section 4.1.1:

“We only kept 82 brain areas from the AAL parcellation, including cortical regions plus the hippocampus and amygdala. We excluded the remaining subcortical regions because source-reconstructed EEG is generally less reliable for deep subcortical structures within this framework, whereas hippocampal and amygdalar sources can be better estimated [11, 12].”

**Comment 1.6:**
*And lastly, I'm not sure if the claims of the model suitability for a broad range of applications are fully supported (chemical neuromodulation, drug delivery, altered states of consciousness; bridges basic and clinical neuroscience). I believe a more grounded framing of the work would not take away from its appeal to the target audience.*

**Response 1.6:**
Thanks for your suggestion. We agree that our original framing was too broad, and we toned down the application claims in the Discussion. In the revised manuscript, we focus the scope on two grounded applications that are directly supported by our results: (i) neuromodulation-related changes in excitability (as illustrated by the wake/NREM simulations) and (ii) neurodegeneration, where E/I balance and brain rhythms are known to play an important role.

The applications we considered in our model were mentioned in different parts of the Discussion:

Discussion:

“This model constitutes an improvement combining ISP mechanisms and a multi-frequency generator of EEG-like dynamics. The ISP control loop can be used as a mechanism for testing alterations in E/I balance associated, for example, with neurodegenerative disorders or during chemical neuromodulation. Using this version of the Jansen-Rit model, we simultaneously reproduced the EEG power spectrum and fMRI FC during wakefulness and sleep.

[...]

Our model is compatible with others, showing how the modulation of excitability, driven by chemical neuromodulation [6, 8, 9], can produce shifts between different regimes (slower and faster brain dynamics). The tuning of model excitability reproduced both EEG spectral slowing and reduced fMRI FC observed during NREM sleep [8, 9].

[...]

In addition, β/γ alterations are consistently reported in brain disorders, including neurodegenerative conditions. The ISP mechanism, combined with the multi-frequency model, allows us to simulate regimes of hypo- and hyperexcitability linked to E/I imbalance [1, 13-15]. [...] our model can potentially simulate shifts from early-stage hyperexcitability and hyperconnectivity toward later hypoexcitable, the increased of slower oscillations relative power, reflecting the progression from preclinical to symptomatic stages of dementia [16, 17].”

**Comment 1.7:**
*- section 2.2 refers to node-level PSP (abbreviation defined later), but it is not clear how that is defined in the multifrequency model (weighted sum of )*

**Response 1.7:**
Thank you for raising this issue. We PSP traces, we mean “EEG traces” (change applied in the new manuscript).

In section 4.4 in Methods, we clarified how the EEG signal is computed in this model:

Methods. Section 4.4:

“The model EEG-like signal can be computed as:

$$\mathrm{EEG}(t)=x_{1}(t)-x_{2}(t)$$

…”

**Comment 1.8:**
*- the $x_1,i$ and $x_2,i$ PSPs are not defined and from the text it is not clear how they are computed exactly.. Similarly, $r^\alpha$ is not appearing in the provided equations.*

**Response 1.8:**
Thanks for remarking on this missing information. We have now defined these state variables in the Methods section:

Methods. Section 4.4:

“The state variables *x_0_*(*t*), *x_1_*(*t*), and *x_2_*(*t*) are defined as a weighted contribution of α and λ subpopulations:

$x_{0}\left( t \right)=r^{\alpha}x_{0}^{\alpha}(t)-{(1-r}^{\alpha})x_{0}^{\gamma}(t)$

$x_{1}\left( t \right)=r^{\alpha}x_{1}^{\alpha}(t)-{(1-r}^{\alpha})x_{1}^{\gamma}(t)$

$x_{2}\left( t \right)=r^{\alpha}x_{2}^{\alpha}(t)-{(1-r}^{\alpha})x_{2}^{\gamma}(t)$

…”

**Comment 1.9:**
*- in section 2.4, the typesetting of 1-Clarkson's distance is confusing*

**Response 1.9:**
Changed to:

“In Fig. 7, we used a measure of similarity (1 - Clarkson’s distance) as a representation of the goodness of fit for simulated EEG”.

**Comment 1.10:**

*There is a typo at the end of the first paragraph of section 4.2*

**Response 1.10:**
Corrected.

**Comment 1.11:**
*- local connectivity constant C is mentioned in the text, but present in the equations*

**Response 1.11:**
Corrected to:

“Neuron populations were connected via constants *C_1_*, *C_2_*, *C_3_* and *C_4_*, which are scaled by a local connectivity constant, *C* = 135, with *C_1_* = *C*, *C_2_* = 0.8*C*, *C_3_* = 0.25*C*, and *C_4_* = 0.25*C*.”

**3. REVIEWER #2**

**Comment 2.1:**
*This manuscript presents an extended Jansen-Rit whole-brain model that incorporates inhibitory synaptic plasticity (ISP) and multi-frequency cortical subpopulations. The goal is to overcome two limitations of classical neural mass approaches: hyperexcitability and lack of spectral richness. The model is validated across EEG, fMRI, and combined multimodal datasets, including wakefulness and NREM sleep. The work is very interesting; however, some important mechanistic points are unclear, and some claims would benefit from clarification.*

**Response 2.1:**
Thank you for these comments and for the positive feedback. We addressed these points below.

**Comment 2.2:**

*Major point:
1- Fig.2: It is not described in a proper way, in the sense that there are many points that are not clear and missing information:

- In 2A, is there a SNIC bifurcation where the stable limit cycle disappears at the level of the saddle node? If not, what is the mechanism?
- In 2B pitchfork with three unstable branches (maybe central fixed point is stable?) There is a region in the parameter space in which the dynamics of the system may diverge for low positive external input. For example, the system presents an unstable limit cycle which is not surrounded by any stable attractor. Moreover, it is not clear how the upper unstable fixed point disappears.
- In 3C there is the same problem about divergence if the system is initiated above the unstable fixed point.*

**Response 2.2:**

We apologize for not describing or explaining the bifurcation plots more profoundly. We have included a better description in the text and figure legends, identifying also the type of bifurcations that we were able to find. Second, the reviewer may be aware that in a 6- or 7-dimensional system with multiple time scales, the bifurcation diagrams are not always simple to interpret and may contain counterintuitive results, as well as behaviors that are not properly represented or captured. Answering the specific points:

2A: The bifurcation that changes the stability of the limit cycle at p~140 does not coincide with any saddle-node bifurcation. Therefore, it is not an SNIC bifurcation. It is of the fold cycle type (or saddle node of limit cycles).

2B: Indeed, there is a range of p between the saddle-node bifurcation at p=6.62 and the Torus bifurcation at p=111 in which the system does not have a stable attractor. In deterministic numerical simulations, the behavior in this range is a continuous oscillation between what seems to be two attractor ruins, one at x0~0.005 and the other at x0~0.15. We found this behavior to be outside the purpose of the paper; some of its consequences are shown in Fig. S1 (Supplementary Information and see **Response 1.2**).

2C: We are not sure if we understand the point of the reviewer. In this case, at all values of p, there is either one stable fixed point, two stable points, or a limit cycle. Therefore, there are no conditions for divergence.

We have made the next changes in the manuscript:

Results section 2.1:

“Across the bifurcation diagrams in Fig. 2, oscillatory stability is shaped by bifurcations of the limit cycle rather than by a saddle-node on invariant circle mechanism. In Fig. 2A, the stability change of the limit cycle around *p* ≈ 140 corresponds to a fold of cycles (saddle-node of limit cycles). In Fig. 2B, between the saddle-node bifurcation at p = 6.62 and the torus bifurcation at p = 111, deterministic simulations do not converge to a stable attractor and instead display persistent oscillatory switching between two regions of state space (around x0 ≈ 0.005 and x0 ≈ 0.15). Because this regime is not central to the aims of the present work, we only report its consequences in the Supplementary Information (Fig. S1). In Fig. 2C, the system always exhibits a stable attractor (one or two stable fixed points or a stable limit cycle) for the explored input range, and no divergent behavior is observed.”

**Comment 2.3:**
*2- Fig.4C: Why is nothing changing for a wide range of the ISP timescale (also, the scale adopted on the figure is not clear)? Since this is the parameter that was added in the present work, I would expect to see its effect, while in this case to me it seems that just by having a constant instead of the differential equation would have been the same. I believe that this point is not well developed in the manuscript since it is fundamental information.*

**Response 2.3:**
Thank you. We have now clarified in the manuscript that the ISP timescale primarily affects the convergence speed of the feedback inhibition (C4), rather than the steady-state oscillatory regime, which explains why Fig. 4C changes little across a wide range of timescales after convergence. We also added new analyses and text to make this explicit (new Fig. 4E-G, and **Responses 1.3** and **3.12**).

**Comment 2.4:**
*3- The definition and role of the noise in the observed spread in frequency are not mentioned clearly.*

**Response 2.4:**

We apologize for the lack of clarity. We have briefly clarified the role of noise in the manuscript, where we made the following change:

Results section 2.2:

“The spread of the frequency of oscillation, for intermediate values of *r*^α^, might be a consequence of the small stochastic fluctuations to the external input, to mimic background variability in afferent drive. This does not alter the underlying oscillatory regime, but it broadens PSD peaks and can produce a small apparent spread in the dominant frequency estimated from finite-length time series, especially near transitions.”

**Comment 2.5:**
*Minor points:
1- “…from slower to faster” is unclear. In general, the frequency band should be defined at the first occurrence to make it clear.*

**Response 2.5:**
Thanks for the suggestions. We have made the proper corrections in the abstract:

Abstract:

“… from slower (δ, θ and α) to faster (β and λ) oscillations”.

**Comment 2.6:**
*2- Improper definition of neural mass models (Wilson-Cowan, Jansen-Rit…) as whole brain models in the beginning (some whole-brain approaches are network of neural masses)*

**Response 2.6:**
Corrected to:

Introduction:

“Classical neural mass models, for example, the Wilson-Cowan, Jansen-Rit, remain as some of the workhorses for simulating large-scale brain dynamics, in the form of whole-brain models.”

**Comment 2.7:**
*3- They claim that: “Our previous works have demonstrated that incorporating multiple subpopulations with different oscillatory frequencies within a cortical column can enhance spectral richness [7, 14, 15].” Do these models also present the problem of hyperexcitability, or otherwise, which is the main contribution of the current work?*

**Response 2.7:**
Thank you for this comment. The present work builds on our previous multi-frequency Jansen-Rit model, and we revised the Introduction to better clarify the novelty of the current contribution in relation to that earlier work (see **Response 1.1** and **3.2**).

**Comment 2.8:**

*4- In section 2.1 they claim: “The frequency of oscillation is not only dependent on the input p, but also on the target value ρ for the firing rate of the pyramidal population. When ρ is set to 3.5 Hz, oscillations are set very robustly around the α (10 Hz) band”. This sentence to me is misleading. In the specific case, where just on subpopulation is considered, I would expect ρ, the target firing rate, like the oscillation frequency if the word “target” is expected as something to achieve. If I am misunderstanding, I would suggest either changing the name of the parameter or explaining better what it refers to*

**Response 2.8:**
Thank you for this comment. We have now clarified in the Results that ρ is a homeostatic set-point for the time-averaged pyramidal firing rate regulated by ISP, not a target oscillation frequency. We also explicitly state that changing ρ shifts the E/I operating point via adaptive inhibition, which can move the system into regimes with different emergent oscillation frequencies.

Results section 2.1:

“Here, ρ denotes a homeostatic set-point for the time-averaged pyramidal firing rate (regulated by ISP), and should not be interpreted as a target oscillation frequency. Instead, changing ρ shifts the E/I operating point via adaptive inhibition, thereby moving the system across dynamical regimes with different emergent oscillation frequencies.”

**Comment 2.9:**
*5- Fig.3: Not really understanding from the text and the caption what I should see in 3B and 3C (curiosity: why are the frequencies in the gamma and alpha bands appearing in “packages”?). Especially, how should I see that the adjustment of feedback inhibition helps to match the model firing rate?*

**Response 2.9:**
Thank you for raising this comment. We have revised the text and the Fig. 3 caption to clarify what panels B-C show and how they demonstrate ISP regulation. In brief, panel B now reports the target firing rate (ρ) versus the time-averaged firing rate produced by the model in noise-free simulations (showing firing-rate clamping), and panel C the corresponding steady-state feedback inhibition (time-averaged C4) reached by ISP to achieve that clamping. We also clarified that the apparent “packages” in the frequency content (Fig. 3A) arise from the finite frequency resolution of the PSD estimation and, in some parameter ranges, from shifts in the dominant spectral component when multiple peaks coexist. See also **Response 3.3**.

Results. Fig. 3:

“Fig. 3. Single-node dynamic. A) Modulation of oscillatory frequency in single-node simulations. Normalized PSD as a function of α versus γ subpopulation proportion, *r*^α^, in the single-region model. The values shown here are the average of 50 random seeds. Note that PSD estimates are computed on a discrete frequency grid (Welch), so narrow-band ridges may appear step-like with *r*^α^. B) Noise-free simulations with fixed *r*^α^ = 0.5, showing the target firing rate *ρ* versus the time-averaged pyramidal firing rate produced by the model. The ISP is “clamping” the ⟨rate⟩ ≈ *ρ*, which is an indicator of successful feedback inhibition regulation. C) Same simulations as in (B), showing the target firing rate *ρ* versus the time-averaged feedback inhibition, given by the inhibitory-to-excitatory local connectivity parameter *C_4_* (the steady-state value reached by ISP to achieve the clamping in B). D) Peak frequency versus external input for the “fast” λ subpopulation (*r*^α^ = 0) and the “slow” α subpopulation (*r*^α^ = 1). The dashed vertical lines correspond to the traces in E) for *r*^α^ = 1, and F) for *r*^α^ = 0.”

**Comment 2.10:**

*6- In the discussion, the authors claim: “In our framework, the target firing rate acts as a bifurcation control parameter [10]. By adjusting this parameter, the system can be placed either to the left or right of a Hopf bifurcation.”. In my opinion this is too simplistic as a description of what is happening in the system, considering its high dimensionality and the bifurcation diagram that were presented in which we could observe many kinds of bifurcation apart from the Hopf.*

**Response 2.10:**
Thanks for bringing this observation. We toned down this statement in the Discussion section:

Discussion:

“Our model extends the classical Jansen-Rit model by incorporating the ISP to modulate excitability and control the oscillatory regime of each subpopulation. In our framework, the target firing rate is a control/input parameter of the closed-loop system (via ISP) that shifts the operating point (mean firing rate / mean inhibitory gain), thereby moving the system through parameter space [4]. In this sense, *ρ* can move the system across bifurcation boundaries (including Hopf), although the specific boundary depends on the parameter slice being examined.”

**Comment 2.11:**
*7- In the discussion, they claim: “Our approach aligns with previous computational work modeling sleep-like dynamics through chemical neuromodulation, but here we demonstrate that a single mechanistic parameter (target excitability) is sufficient to reproduce multi-modal empirical data [52-54].”. I believe this sentence is a bit exaggerated because, even if it is true that they show that by changing parameter ρ they can fit experimental data, they are not testing sensitivity about other parameters (it seems to me that also the background input p could be a sensitive parameter).*

**Response 2.11:**
Thanks for raising this issue. In the Discussion section, we have toned down this statement and re-focused the implications about our modeling approach in the context of sleep and neuromodulation (see **Responses 1.6** and **3.5**).

**4. REVIEWER #3**

**4.1 Summary and overall evaluation**

**Comment 3.1:**
*Summary:

The authors present a novel extension of the classical Jansen-Rit neural mass model, aiming to increase its flexibility and biological plausibility. In contrast to the standard Jansen-Rit formulation, each neural mass is subdivided into two interconnected subpopulations, expressing fast and slow rhythmic activity, which allows it to switch between them and provide a broader spectrum of frequencies. Furthermore, the authors implement a feedback inhibition control mechanism to adjust inhibitory gain in order to maintain the system near desired dynamical regimes. The model is first analyzed at the single-node level to characterize its intrinsic dynamical repertoire and bifurcation structure. Subsequently, it is embedded into a whole-brain network using source-level functional connectivity derived from EEG recordings of healthy individuals. The authors further apply the model to multimodal EEG-fMRI data acquired during sleep, exploring whether the modified dynamics can capture state-dependent changes in brain activity.*

*Conclusion:

The authors combine two recent extensions of the Jansen-Rit model into a unified framework that offers a flexible and potentially valuable tool for whole-brain modeling. The approach is novel and likely to be helpful for the community, as it improves the dynamical richness and stability of large-scale simulations. The manuscript thoroughly examines the model and provides rigorous validation across various scales and datasets. Meanwhile, the manuscript would benefit from further exploration of the suggested model and a clearer justification of several modeling choices to strengthen confidence in the proposed framework and to provide a more detailed understanding of its advantages and limitations.*

**Response 3.1:**
Thanks for your summary and the positive evaluation. We have addressed all of your concerns as detailed below.

**Comment 3.2:**
*Questions to the Authors:

1. In the Introduction (p. 9), you refer to “hyperexcitability” as a known problem. Could you explain this in more detail? This phenomenon depends strongly on the specific model formulation and fitting strategy, and different approaches exist to address overshooting excitation (as you also note later in the Discussion). Additionally, what is the conceptual link between increased excitation, plasticity, and the feedback-inhibition mechanism introduced later?*

**Response 3.2:**
Thank you for this comment. We revised the Introduction to (i) clarify what we mean by model hyperexcitation/over-saturation, (ii) distinguish this from biological/clinical hyperexcitability, (iii) state explicitly that the onset of this regime is model- and fitting-dependent, and (iv) clarify the conceptual link between increased excitation, ISP, and activity-dependent feedback inhibition as a homeostatic mechanism regulating firing rates/operating points.

We have made the following changes to the manuscript:

Introduction:

“A key challenge, however, lies in setting these models to reflect biologically plausible excitation/inhibition (E/I) balance, especially in whole-brain simulations where runaway excitation (hereafter ‘hyperexcitation’ or over-saturation) can dislocate realistic network behavior [4, 5, 18]. This over-saturation, manifested as increased excitatory inputs to neuronal populations, can saturate the model input-output function, disrupting oscillatory activity in biophysical models.

[...]

Here, we use the term “model hyperexcitability” to refer to this behavior when the bifurcation occurs with respect to an external input parameter, specifically, when it causes the over-saturation of the input-output function of the neural mass models. This behavior is particularly observed in large-scale networks with increased inter-regional coupling, where excessive effective excitation (often due to stronger coupling and/or parameter combinations) drives the neural mass input-output sigmoid into saturation, thereby collapsing oscillations toward a fixed point [4, 5, 7, 18].

[...]

This should not be conflated with biological/clinical hyperexcitability, which denotes a pathophysiological increase in neuronal excitability and/or E/I imbalance in vivo. We use the qualifier “model hyperexcitation” to avoid ambiguity.

[...]

Previous works have suggested that homeostatic inhibitory synaptic plasticity (ISP) can counteract model hyperexcitation by implementing activity-dependent feedback inhibition. The inhibitory strength is adjusted online to stabilize mean firing rates (and thus regional operating points), preventing saturation and helping maintain sustained oscillations and network-wide integration [1, 4, 5].

[...]

The conditions under which a given whole-brain model enters this saturated, non-oscillatory regime are model- and fitting-dependent. One advantage of the ISP dynamical mechanism, compared to heuristic approaches [6, 7], is that it does not require a priori knowledge of how the model’s many interacting parameters jointly modulate excitability.”

**Comment 3.3:**
*2. You state that the two subpopulations are tuned to oscillate on fast limit cycles in either the alpha or gamma range. What are the corresponding slow-limit-cycle frequencies for both subpopulations? In the classical Jansen-Rit model, the slow cycle typically lies in the theta/delta range—how is this preserved or modified for the population tuned to gamma?*

**Response 3.3:**
Thank you for this comment. We added a short paragraph in the Results (Single-node dynamics; Fig. 3D-F) reporting the slower limit-cycle frequencies for both subpopulations.

Results. Section 2.1:

“Finally, we examined the “slower” limit-cycle frequencies for each subpopulation, in addition to the fast α/γ regimes. For both the α (*r*^α^ = 1) and γ (*r*^α^ = 0) subpopulations, the model exhibits two oscillatory regimes depending on external input *p* (Fig. 3D-F). At lower input (*p* = 120; slower regime), the α subpopulation shows the expected low-frequency limit cycle with a peak around ~3 Hz (δ/θ range), consistent with the classical Jansen-Rit slow cycle. The γ subpopulation also presents a slower limit cycle, but with a higher peak frequency around ~20 Hz (β range). At higher input (*p* = 300; fast regime), the α and γ subpopulations express their respective fast limit cycles (α ~10 Hz; γ >30 Hz), indicating that the γ tuning shifts both fast and slow oscillatory regimes while preserving the presence of a distinct slower cycle.”

Results. Fig. 3:

“Fig. 3. Single-node dynamic. A) Modulation of oscillatory frequency in single-node simulations. Normalized PSD as a function of α versus γ subpopulation proportion, *r*^α^, in the single-region model. The values shown here are the average of 50 random seeds. Note that PSD estimates are computed on a discrete frequency grid (Welch), so narrow-band ridges may appear step-like with *r*^α^. B) Noise-free simulations with fixed *r*^α^ = 0.5, showing the target firing rate *ρ* versus the time-averaged pyramidal firing rate produced by the model. The ISP is “clamping” the ⟨rate⟩ ≈ *ρ*, which is an indicator of a successful feedback inhibition regulation. C) Same simulations as in (B), showing the target firing rate *ρ* versus the time-averaged feedback inhibition, given by the inhibitory-to-excitatory local connectivity parameter *C_4_* (the steady-state value reached by ISP to achieve the clamping in B). D) Peak frequency versus external input for the “fast” λ subpopulation (*r*^α^ = 0) and the “slow” α subpopulation (*r*^α^ = 1). The dashed vertical lines correspond to the traces in E) for *r*^α^ = 1, and F) for *r*^α^ = 0.”

**Comment 3.4:**
*3. The use of two subpopulations per cortical column is a pragmatic strategy to broaden the frequency repertoire; however, EEG frequency-band origins remain an active area of debate and exhibit regional variability. Could you provide a more detailed justification for reducing the origin of different brain rhythms to the same two subpopulations in each cortical column and discuss the implications of this simplification?*

**Response 3.4:**

Thank you for pointing at this issue. We agree that EEG generator rhythms remain debated and can vary across brain regions. We have clarified now in the manuscript that using two subpopulations per cortical column is a parsimonious modeling choice, aimed at capturing different oscillatory rhythms in cortical layers (Methods). This approach has been previously used in other whole-brain phenomenological, models (<https://doi.org/10.1016/j.neuroimage.2017.03.023>. Finally, we also acknowledge the simplification of our approach in the Discussion section of the new manuscript.

Discussion:

“We used two rhythm-generating subpopulations in every cortical region. Although this is a parsimonious abstraction to increase spectral richness [19], it does not aim to provide detailed mechanisms, and alternative choices can be used to shape oscillatory frequencies (e.g., E/I loop delays, synaptic time constants/receptor kinetics, and axonal transmission delays). They are not explicitly modeled here and can be incorporated in future extensions.”

Methods:

“The rationale of combining those to subpopulations is grounded in the organization of cortical layers, where different neuronal subpopulations exhibit distinct input-output dynamics and generate frequency-specific rhythms [20]. Superficial layers tend to produce faster oscillations, such as β and γ, while deeper and granular layers are associated with slower α and θ activity [20]. Here, we use two subpopulations as a parsimonious modeling choice to broaden the nodal spectral repertoire while keeping the whole-brain model tractable for fitting. This does not mean that this approach should be treated as an account of the unique origin of each rhythm in every region. Similar multi-timescale approaches have been explored previously in whole-brain modeling to increase spectral richness at each node [21]. In our model, the EEG reflects a weighted summation of these laminar contributions, and the resulting broadband spectrum emerges from the combined activity of spatially and synaptically distinct rhythm-generating circuits.”

**Comment 3.5:**
*4. Please clarify which empirical or theoretical phenomena specifically require extending the model to higher frequencies. The classical Jansen-Rit model can already produce alpha, theta, and delta rhythms (although just within a restricted parameter range), which are also the primary rhythms relevant for, e.g., the sleep experiment included in this study. Therefore, a brief review of why beta and gamma frequencies are important, such as in plasticity and learning, would provide helpful context.*

**Response 3.5:**
Than you. We now addressed this point in the Discussion by clarifying why extending the model to β/γ is relevant beyond the sleep experiment (where slow rhythms dominate).

Discussion:

“While the classical Jansen-Rit model can reproduce δ/θ/α rhythms, this typically occurs within restricted parameter regimes and does not capture the full-band spectral structure. Extending the model to support β/γ dynamics broadens its dynamical repertoire, opening the door to study phenomena where higher frequencies are central. For example, the model can be used to explore how attention and working memory, as well as perceptual integration/binding, are related to β/γ power modulation [22]. In addition, β/γ alterations are consistently reported in brain disorders, including neurodegenerative conditions. The ISP mechanism, combined with the multi-frequency model, allow to simulate regimes of hypo- and hyperexcitability linked to E/I imbalance [1, 13-15]. Prior studies have used PET-based amyloid and tau deposition to modulate local excitability and mimic E/I imbalance [14, 23]. Our model can potentially simulate shifts from early-stage hyperexcitability and hyperconnectivity toward later hypoexcitable, spectrally slowed states, reflecting the progression from preclinical to symptomatic stages of dementia [16, 17]. Further, both EEG and fMRI features can be captured, providing a multimodal modeling tool for exploring how brain excitability modulates brain function across diverse contextual and clinical conditions.”

**Comment 3.6:**
*5. Regarding Results 2.2: Why are only the amygdala and hippocampus included among subcortical regions?*

**Response 3.6:**
Thank you for this comment. We only kept 82 brain areas from the AAL parcellation, including cortical regions plus the hippocampus and amygdala. This choice was motivated by the fact that source-reconstructed EEG signals are generally less reliable for deep subcortical structures, whereas hippocampal and amygdalar sources can be estimated more robustly:

<https://www.nature.com/articles/s41467-019-08665-5>

<https://academic.oup.com/braincomms/article/5/1/fcad023/7030489>

We have made the following changes to the manuscript:

Methods. Section 4.1.1:

“We only kept 82 brain areas from the AAL parcellation, including cortical regions plus the hippocampus and amygdala. We excluded the remaining subcortical regions because source-reconstructed EEG is generally less reliable for deep subcortical structures within this framework, whereas hippocampal and amygdalar sources can be better estimated [11, 12].”

**Comment 3.7:**
*6. Also in 2.2: While it is intuitive that high p leads to slower oscillations, why do high values of p also produce slower dynamics? A brief explanation referencing the bifurcation structure described in Section 2.1 might help to understand this behavior better.*

**Response 3.7:**

The reviewer raises an interesting observation. A possible answer can be found in the additional bifurcation plots that we are now including after the concerns of other reviewers. As shown in Fig. S1 (Supplementary Information), the transition from the bifurcation diagram obtained with target=2.5 to that of target=3.5 is quite complicated. The shift from supercritical to subcritical Hopf bifurcation is first accompanied by the existence of a range of input p in which the periodic orbit is stable, then unstable, and finally stable again by a succession of torus bifurcations. This creates a range of p values where a complicated landscape of periodic orbits exists. This landscape is not evident in the bifurcation diagram, but its consequences can be appreciated in numerical simulations when p ~ 100-150. Thus, the Hopf bifurcation creates a stable orbit that upon further increases in p falls into this complex orbit regime (or maybe its ruins), causing a slowing down of the oscillation. A further increase in p takes the system away from this, making the oscillations robust again.

**Comment 3.8:**
*7. In Section 2.3, canonical EEG bands appear to be characterized solely by their frequency ranges. However, in fact, they are also defined by characteristic amplitudes, spatial topographies, and temporal dynamics (e.g., alpha amplitude fluctuations, occipital dominance, and a typical amplitude of 20-60 µV). Since the original Jansen-Rit model captures some of these aspects, to what extent does your modified model reproduce these additional features?*

**Response 3.8:**
This is a fair concern, and you are correct regarding canonical EEG bands are not defined solely by frequency ranges. We added now an explicit qualitative illustration in the Results (Section 2.3; new Fig. 7) showing band-limited simulated EEG time series and α-band envelope dynamics for the ISP-fit model. We clarified in the Discussion (Limitations) that reproducing these additional features (e.g., µV scaling, occipital alpha dominance, and detailed temporal structure) is out of scope for this work, and would require additional forward modeling/calibration and further physiological constraints.

Results. Section 2.3:

“To illustrate the qualitative temporal structure of the ISP-fit model beyond FC metrics, we additionally report representative EEG time series and amplitude-envelopes for the best-fitting parameter set (*K* = 0.675, *r*^α^ = 0.575, *ρ* = 2.5 Hz) (Fig. 7). We show a 10-s segment of simulated EEG, band-pass filtered into canonical frequency bands (δ-γ). We also plot the α-band envelope from the same simulation, which captures the slow α-band power fluctuations (amplitude dynamics).”

Results. Fig. 7:

“Fig. 7. Band-limited model time series and relative band power from the ISP-fit (K = 0.675, *r*^α^ = 0.575, *ρ* = 2.5 Hz). Representative 10-s segment of simulated EEGs, band-pass filtered into canonical frequency bands: δ (0.5-4 Hz), θ (4-8 Hz), α (8-13 Hz), β (13-30 Hz), and γ (30-40 Hz). The bottom panel shows the EEG α band envelope (α band power fluctuations).”

Discussion:

“While the model can reproduce oscillations across canonical frequency bands and captures alpha-band power fluctuations at the whole-brain level, we did not aim to match additional defining properties of canonical rhythms such as absolute amplitudes (e.g., µV scaling), spatial topographies (e.g., occipital dominance of alpha), or the full temporal structure across subjects and conditions. Reproducing these features would require additional calibration steps and potentially further physiological constraints, which are beyond the scope of this study. Also, while the model reproduces state-dependent spectra and connectivity, exemplary wake/sleep time series can appear less regular than canonical alpha or highly synchronized delta rhythms. This is partly due to limitations in reproducing the slowest EEG components in resting-state simulations, which we did not explicitly optimize.”

**Comment 3.9:**
*8. In 2.3, why do you consider only delta, theta, alpha, and beta bands for EEG-derived FC, even though one of the motivations for the multi-frequency model is to include gamma oscillations?*

**Response 3.9:**
Thanks for your comment. In our study, the empirical EEG data were filtered up to 40 Hz during preprocessing, which makes the γ band only partially represented and strongly affected by filter roll-off near the upper cutoff. For this reason, we restricted the analyses in Sections 2.3 and 2.4 to δ-β bands.

**Comment 3.10:**
*9. Please justify the choice of SSIM as the similarity metric. Why was it preferred over other metrics, and could you also provide correlation coefficients to offer readers a clearer sense of similarity magnitude?*

**Response 3.10:**
We appreciate your suggestion. We chose SSIM because it jointly captures similarity in the overall connectivity structure and in the absolute FC level (i.e., it is sensitive to both the spatial pattern and mean connectivity) (add cites here). By contrast, Pearson’s correlation primarily reflects pattern similarity and can remain high even when the simulated FC differs from the empirical FC in overall connectivity strength. We now also report Pearson’s correlation coefficients in Fig. 6. This comparison shows that Pearson’s *r* can be relatively high (e.g., >0.5 in the single-frequency model) while SSIM remains low and the corresponding FC matrices appear qualitatively dissimilar to the empirical FC, supporting our use of SSIM as the primary fitting metric.

Results. Fig. 6:

“Fig. 6. Comparison of the single- and multi-frequency models in fitting empirical EEG FC. A) The structural similarity index (SSIM) was used to measure the goodness of fit (SSIM = 1, perfect fit). B) Complementary to SSIM, we reported Pearson’s correlation between simulated and empirical matrices (r = 1, perfect fit). The columns represent different EEG frequency bands. C) Simulated EEG FCs from the single-frequency model. D) Simulated FCs from the multi-frequency model. E) Empirical EEG FC matrices. ***: |Cohen’s D| > 1.2. Each point corresponds to different models’ realizations (50 random seeds).”

**Comment 3.11:**
*10. Why do the analyses in 2.3 rely exclusively on FC metrics? Could additional metrics, such as FCD or frequency spectra, be incorporated, mainly since spectral fitting is used later in the study?*

**Response 3.11:**
We appreciate your observation. In Section 2.3, we focused on FC-based metrics to directly test whether the multi-frequency + ISP mechanism enables reproducing band-limited EEG connectivity across a broad parameter space. We agree that additional targets such as FCD and/or explicit spectral constraints could be incorporated. However, adding this analysis is out of scope, and would substantially increase the complexity of the fitting problem potentially masking the parameter space. A systematic multi-objective fitting (e.g., FC and FCD) is trully beyond the scope of this work.

**Comment 3.12:**
*11. Relatedly, you state that the system is “unable to reach” certain areas of parameter space without feedback inhibition. Would these areas become accessible if an additional fitting target were included—such as a synchrony-related metric that counteracts the bias toward hypersynchronized regimes, usually mainly driven by the genuine similarity between functional and structural connectivity? In Section 2.4, you note to yourselves that additional targets (e.g., a spectral constraint) can "mask" parts of the parameter space.*

**Response 3.12:**
We appreciate your point. In our case, the “inaccessible” regions without ISP primarily reflect a dynamical limitation rather than an optimization bias or the absence of additional fitting targets. Additional fitting targets (e.g., synchrony- or spectrum-related constraints) can influence which solutions are selected and can mask parts of the parameter space, but they cannot recover regions where the underlying dynamics do not support sustained oscillatory activity. We clarified this point in the Results section.

Results. Section. 2.3:

“The model without ISP is characterized by a parameter space with a small region of correlated activity (Fig. 5A). It is unable to reach correlated activity before being hyperexcited, as no compensatory mechanisms were introduced in the form of ISP. Further, it cannot generate correlated activity when mixing α and γ subpopulations in cortical columns; that is, near 0 mean FC for *r*^α^ values around 0.5 suggests model frustration (inability to generate correlated activity). This limitation reflects the local and network dynamics of the model without ISP: for large portions of the (K, *r*^α^) space, increased effective excitation drives the system toward saturation and loss of sustained oscillations, which prevents the emergence of stable band-limited envelope fluctuations and, consequently, correlated activity. Therefore, the restricted parameter region in Fig. 5A is primarily due to the model’s inability to sustain oscillatory dynamics without an adaptive inhibitory mechanism, rather than a bias induced by additional fitting targets or anatomical priors. Including ISP in the model with *ρ* = 2.5 Hz, enriches the parameter landscape with a wider range of parameters where connectivity correlations can emerge, from hypo to hyperconnectivity patterns, including intermediate connectivity regimes of activity (Fig. 5B). Interestingly, correlations for intermediate values of *r*^α^ are possible to achieve when using ISP. Increasing global coupling in this scenario produced multi-frequency EEG FC connectivity, with maximal SSIM values in the proximity of *r*^α^ = 0.5 and *K* = 0.5.”

**Comment 3.13:**
*12. Fig. 6: Panels C and D appear without labels; please add the corresponding letters to match the caption.*

**Response 3.13:**
Thank you. Corrected.

**Comment 3.14:**

*13. In Section 2.4, why are the fitting targets limited to EEG frequency and fMRI-derived FC? Why not use spectral and FC targets from both EEG and fMRI?*

**Response 3.14:**
Thanks for your comment. Our fitting targets in Section 2.4 were intentionally restricted to EEG spectral features and fMRI-derived FC to keep the multimodal optimization tractable and aligned with the main goal of this work. Further, we choose not to include the EEG FC because source reconstruction might be unreliable (the original data has 32 EEG channels). We agree that extending the fitting procedure to include additional targets from both modalities (e.g., combined EEG spectral + FC constraints and fMRI spectral/temporal constraints) is a natural next step, but this was beyond the scope of the present manuscript. We have acknowledged this in the Discussion (Limitations)

Discussion:

“Fifth, the current implementation is limited to resting-state EEG and fMRI. Extending the model to simulate task-evoked activity, e.g., task-evoked β-γ changes, would help explore context-dependent changes in connectivity and cognitive processing [6, 24]. Sixth, we primarily defined canonical EEG bands by their frequency ranges and focused on spectral content and large-scale connectivity validation (EEG functional connectivity across bands and fMRI FC). Accordingly, we did not include additional fitting targets from both modalities (e.g., simultaneous EEG spectral and FC constraints together with fMRI spectral/temporal constraints), which would represent a future extension of the model”.

**Comment 3.15:**
*14. For Fig. 8, please provide the correlations between empirical and simulated FCs, and comment on the degree of inter-individual variability.*

**Response 3.15:**
We appreciate your comment. We have clarified now that the results in Fig. 8 are based on group-averaged empirical FC (and simulations averaged across seeds), and we did not perform subject-specific model fitting or individual-level simulations in this study, which is beyond the scope of the present work. Because the analyses were conducted at the group level, we did not quantify inter-individual variability here. Regarding FC correlations, we now report Pearson's correlation in addition to SSIM (see **Response 3.10**).

Results. Section 2.4:

“For wakefulness, the best fit was found for higher coupling and medium firing rates (*K* = 1.44, *ρ* = 2.56 Hz, SSIM = 0.34, Pearson’s *r* = 0.45, Fig. 8A). In contrast, N3 sleep was better characterized by low coupling and low firing rates (*K* = 0.52, *ρ* = 2.1 Hz, SSIM = 0.29, Pearson’s *r* = 0.38, Fig. 8B).”

**Comment 3.16:**

*15. Fig. 8C: The (exemplary) simulated wake signal does (from first view) seem not to resemble typical alpha activity (as it is too irregular), and the deep-sleep signal also lacks the highly regular, synchronized delta waves observed empirically. How is this in the empirical dataset used here? Please comment on the model's ability to capture these qualitative features and provide the corresponding empirical examples of time series for comparison.*

**Response 3.16:**
Thanks for raising this concern. We added the corresponding empirical wake and N3 time series examples for comparison in Fig. 9. We also have now addressed this point in the Discussion (Limitations) (see **Response 3.8**)

Results. Fig. 9:

“Fig. 9. Example simulations for the EEG and fMRI sleep dataset. A) Empirical fMRI FC matrices for wakefulness and N3 stages. B) Best simulated matrices. C) Empirical EEG traces (for one representative participant). D) Averaged empirical EEG power spectrum. E) Examples of EEG time courses from simulations. F) Simulated EEG power spectrum. The model’s parameters were *K* = 1.44 and *ρ* = 2.56 Hz, for wakefulness, and *K* = 0.52, *ρ* = 2.1 Hz, for N3 sleep. The values shown here are the average of 50 random seeds.”

**Comment 3.17:**

*16. Fig. 8D: In NREM stage 3, please include the empirical spectra for comparison, as empirical power spectra typically show narrow-band, high-amplitude delta dominance*

**Response 3.17:**
Thanks for your comment. Now we have included the empirical power spectra for NREM stage 3 for direct comparison in Fig. 9. We also note in the Discussion that the model has limitations in reproducing the typical narrow-band, high-amplitude delta dominance observed empirically in N3 power spectra (see **Responses 3.8** and **3.16**)

**Comment 3.18:**
*17. On page 14, “Janse-Rit” should be corrected to “Jansen-Rit.”*

**Response 3.18:**
Thank you. Corrected

**Comment 3.19:**
*18. On page 14, you state: "Superficial layers tend to produce faster oscillations such as α and θ, while deeper and granular layers are associated with slower β and γ activity." In how far are alpha and theta faster than beta and gamma?*

**Response 3.19:**
Thanks for pointing out this mistake. We already have corrected this statement:

Methods. Section 4.4:

“…faster oscillations such as β and γ, while deeper and granular layers are associated with slower θ and α activity”.

**Comment 3.20:**
*19. Page 14 again states that the model can express frequencies from delta to gamma. Why, then, are gamma-band results not included in Sections 2.3 and 2.4?*

**Response 3.20:**
Thank you for pointing out this issue. We already have clarified this methodological choice in **Response 3.9.**

**References**

1.	Coronel‐Oliveros C, Gómez RG, Ranasinghe K, Sainz‐Ballesteros A, Legaz A, Fittipaldi S, et al. Viscous dynamics associated with hypoexcitation and structural disintegration in neurodegeneration via generative whole‐brain modeling. Alzheimer's & Dementia. 2024. doi: 10.1002/alz.13788.

2.	Otero M, Lea-Carnall C, Prado P, Escobar M-J, El-Deredy W. Modelling neural entrainment and its persistence: inﬂuence of frequency of stimulation and phase at the stimulus oﬀset. Biomedical Physics & Engineering Express. 2022. doi: 10.1088/2057-1976/ac605a.

3.	Jansen BH, Rit VG. Electroencephalogram and visual evoked potential generation in a mathematical model of coupled cortical columns. Biological Cybernetics. 1995;73(4):357-66. doi: 10.1007/BF00199471.

4.	Stasinski J, Taher H, Meier JM, Schirner M, Perdikis D, Ritter P. Homeodynamic feedback inhibition control in whole-brain simulations. PLOS Computational Biology. 2024;20(12):e1012595.

5.	Abeysuriya RG, Hadida J, Sotiropoulos SN, Jbabdi S, Becker R, Hunt BAE, et al. A biophysical model of dynamic balancing of excitation and inhibition in fast oscillatory large-scale networks. PLOS Computational Biology. 2018;14(2):e1006007-e. doi: 10.1371/journal.pcbi.1006007.

6.	Coronel-Oliveros C, Gießing C, Medel V, Cofré R, Orio P. Whole-brain modeling explains the context-dependent effects of cholinergic neuromodulation. NeuroImage. 2023;265:119782-. doi: 10.1016/j.neuroimage.2022.119782.

7.	Herzog R, Mediano PA, Rosas FE, Luppi AI, Sanz-Perl Y, Tagliazucchi E, et al. Neural mass modeling for the masses: Democratizing access to whole-brain biophysical modeling with FastDMF. Network Neuroscience. 2024;8(4):1590-612.

8.	Deco G, Hagmann P, Hudetz AG, Tononi G. Modeling Resting-State Functional Networks When the Cortex Falls Asleep: Local and Global Changes. Cerebral Cortex. 2014;24(12):3180-94. doi: 10.1093/cercor/bht176.

9.	Lehue F, Coronel-Oliveros C, Medel V, Liebe T, Kaufmann J, Orellana S, et al. Noradrenaline and acetylcholine shape functional connectivity organization of NREM substages: An empirical and simulation study. PLOS Computational Biology. 2025;21(10):e1012852.

10.	Goldman JS, Kusch L, Aquilue D, Yalçınkaya BH, Depannemaecker D, Ancourt K, et al. A comprehensive neural simulation of slow-wave sleep and highly responsive wakefulness dynamics. Frontiers in Computational Neuroscience. 2023;16. doi: 10.3389/fncom.2022.1058957.

11.	Unnwongse K, Rampp S, Wehner T, Kowoll A, Parpaley Y, von Lehe M, et al. Validating EEG source imaging using intracranial electrical stimulation. Brain Communications. 2023;5(1):fcad023.

12.	Pizzo F, Roehri N, Medina Villalon S, Trébuchon A, Chen S, Lagarde S, et al. Deep brain activities can be detected with magnetoencephalography. Nature communications. 2019;10(1):971.

13.	Kudo K, Ranasinghe KG, Morise H, Syed F, Sekihara K, Rankin KP, et al. Neurophysiological trajectories in Alzheimer’s disease progression. Elife. 2024;12:RP91044.

14.	Ranasinghe KG, Verma P, Cai C, Xie X, Kudo K, Gao X, et al. Altered excitatory and inhibitory neuronal subpopulation parameters are distinctly associated with tau and amyloid in Alzheimer’s disease. eLife. 2022;11. doi: 10.7554/eLife.77850.

15.	Fischer MHF, Zibrandtsen IC, Høgh P, Musaeus CS. Systematic review of EEG coherence in Alzheimer’s disease. Journal of Alzheimer’s Disease. 2023;91(4):1261-72.

16.	Martínez-Cañada P, Perez-Valero E, Minguillon J, Pelayo F, López-Gordo M, Morillas C. Combining Aperiodic 1/f Slopes and Brain Simulation: An EEG/MEG Proxy Marker of Excitation/Inhibition Imbalance in Alzheimer’s Disease. bioRxiv. 2023. doi: 10.1101/2022.12.21.521529.

17.	Maestú F, de Haan W, Busche MA, DeFelipe J. Neuronal excitation/inhibition imbalance: core element of a translational perspective on Alzheimer pathophysiology. Ageing Research Reviews. 2021;69:101372-. doi: 10.1016/j.arr.2021.101372.

18.	Deco G, Ponce-Alvarez A, Hagmann P, Romani GL, Mantini D, Corbetta M. How Local Excitation-Inhibition Ratio Impacts the Whole Brain Dynamics. Journal of Neuroscience. 2014;34(23):7886-98. doi: 10.1523/JNEUROSCI.5068-13.2014.

19.	Deco G, Cabral J, Woolrich MW, Stevner ABA, van Hartevelt TJ, Kringelbach ML. Single or multiple frequency generators in on-going brain activity: A mechanistic whole-brain model of empirical MEG data. NeuroImage. 2017;152:538-50. doi: 10.1016/j.neuroimage.2017.03.023.

20.	Maier A, Adams GK, Aura C, Leopold DA. Distinct superficial and deep laminar domains of activity in the visual cortex during rest and stimulation. Frontiers in systems neuroscience. 2010;4:31.

21.	Mendoza-Halliday D, Major AJ, Lee N, Lichtenfeld MJ, Carlson B, Mitchell B, et al. A ubiquitous spectrolaminar motif of local field potential power across the primate cortex. Nature Neuroscience. 2024;27(3):547-60.

22.	Fries P. Rhythms for cognition: communication through coherence. Neuron. 2015;88(1):220-35.

23.	Patow G, Stefanovski L, Ritter P, Deco G, Kobeleva X, Initiative AsDN. Whole-brain modeling of the differential influences of amyloid-beta and tau in Alzheimer’s disease. Alzheimer's Research & Therapy. 2023;15(1):210.

24.	Pfeffer T, Ponce-Alvarez A, Tsetsos K, Meindertsma T, Gahnström CJ, van den Brink RL, et al. Circuit mechanisms for the chemical modulation of cortex-wide network interactions and behavioral variability. Science Advances. 2021;7(29). doi: 10.1126/sciadv.abf5620.
