# Supplementary material for: A multi-frequency whole-brain neural mass model with homeostatic feedback inhibition
Source: PLoS Comput Biol. 2026 May 13;22(5):e1013463. doi: 10.1371/journal.pcbi.1013463 (PMC13183287; doi:10.1371/journal.pcbi.1013463)
Supplement: Attachment — Submitted filename: Response_R2.docx [file pcbi.1013463.s007.docx]

**RESPONSE TO EDITORS AND REVIEWERS**

**Manuscript ID:** PCOMPBIOL-D-25-01699
**Title:** *A multi-frequency whole-brain neural mass model with homeostatic feedback inhibition*

Dear Drs. van Albada and Berry,

Thank you and the Reviewers for the careful evaluation of our manuscript. Below, we provide a point-by-point response to all editorial and Reviewer comments. The changes in the manuscript are in red. All modifications in the revised manuscript are tracked in the “Revised Manuscript with Track Changes” file.

We look forward to the evaluation of this new version.

Best wishes,

Agus Ibáñez

**1. EDITOR / JOURNAL REQUIREMENTS**

**Comment E1:**
*Please ensure that the CRediT author contributions listed for every co-author are completed accurately and in full. At this stage, the following Authors/Authors require contributions: Carlos Coronel-Oliveros, Josephine Cruzat, Marilyn Gatica, Raul Gonzalez-Gomez, Hernán Hernandez, Rubén Herzog, Natalia Kowalczyk-Grębska, Fernando Lehue, Vicente Medel, Iván Mindlin, Patricio Orio, Pavel Prado, Enzo Tagliazucchi, and Agustín Ibáñez. Please ensure that the full contributions of each author are acknowledged in the "Add/Edit/Remove Authors" section of our submission form. The list of CRediT author contributions may be found here:*

[*https://journals.plos.org/ploscompbiol/s/authorship#loc-author-contributions*](https://journals.plos.org/ploscompbiol/s/authorship#loc-author-contributions)

**Response E1:**

We have now completed all CRediT roles for every co-author in the submission system and mirrored them in the manuscript.

**2. REVIEWER #1**

**Comment 1.1:**
 I'd like to thank the authors for the revision; my comments were appropriately addressed.

**Response 1.1:**
Thanks for the positive feedback.

**Comment 1.2:**

I have one minor comment regarding the fitting of EEG source data. There is no need to reduce the size of the simulated system for fitting EEG data features derived from a subset of the nodes. However I believe the results would not change qualitatively in present work, the authors may take this into consideration either in the discussion (if additional revisions are made), or in future work.

**Response 1.2:**

Thanks for this suggestion. We add this consideration in the Discussion section as follows:

Discussion:

“[...] In addition, although EEG-derived features can be fitted using only a subset of observed nodes without reducing the size of the simulated system, we adopted a reduced fitting setup for computational convenience; this simplification is not expected to qualitatively affect the present results, but a full-system fitting strategy should be considered in future work.”

**3. REVIEWER #2**

**Comment 2.1:**
In this resubmitted version, the authors addressed most of the concerns. A few points remain to be improve:

**Response 2.1:**
Thanks for the positive evaluation. All your concerns have been addressed below.

**Comment 2.2:**

The authors deflected this rather than answered it directly. The letter politely but clearly asks for one or two sentences clarifying whether the prior multi-frequency model (without ISP) also suffered from hyperexcitability, a simple but scientifically important clarification.

**Response 2.2:**

We apologize for the lack of clarity. Both the classical Jansen-Rit model and multi-frequency implementations without ISP can enter hyperexcitable or over-saturated regimes in whole-brain simulations, because they lack an adaptive mechanism to dynamically regulate feedback inhibition. By contrast, the multi-frequency model used in our previous dementia work already incorporated ISP precisely to mitigate this problem. Therefore, the present manuscript does not introduce a new model, but rather provides a systematic characterization of that previously used framework, including its bifurcation structure, the effects of key parameters on oscillatory regimes, and its validation against EEG and fMRI data across wakefulness and NREM sleep. We have revised the manuscript to make this distinction explicit and to avoid overstating model novelty:

Abstract:

“Here, we thoroughly characterize a previously introduced multi-frequency Jansen-Rit neural mass model with inhibitory synaptic plasticity (ISP) aimed at overcoming these limitations. The ISP adjusts inhibitory feedback onto pyramidal neurons to clamp their firing rates around a target value.”

Introduction:

“Classical neural mass models, such as the Wilson-Cowan, Jansen-Rit, remain as some of the workhorses for simulating large-scale brain dynamics, in the form of whole-brain models [1]. These models undergo bifurcations, such as Hopf bifurcations [2, 3], beyond which node dynamics converge to stable fixed points and cease to oscillate. This transition disrupts oscillatory coherence and prevents the emergence of globally correlated network activity. Both the classical Jansen-Rit models [4], as well as multi-frequency extensions [5], can enter hyperexcitable or over-saturated regimes in whole-brain simulations. Here, we use the term “model hyperexcitability” to refer to this behavior: when the bifurcation occurs with respect to an external input parameter, specifically, when it causes the over-saturation of the input-output function of the neural mass models.”

“Here, we study a previously introduced multi-frequency neural mass model used in dementia [5, 6], based on the Jansen-Rit equations [4, 5], that includes biologically motivated inhibitory synaptic plasticity (ISP) mechanism [7, 8].”

Discussion:

“In this work, we systematically characterized and validated a semi-empirical Jansen-Rit whole-brain model able to simulate both EEG and fMRI BOLD dynamics from biologically plausible mechanisms.”

**Comment 2.3:**

The overclaim was removed, but the sensitivity question was never addressed. A one-sentence acknowledgment that p was held fixed in the sleep/wake comparison would be useful.

**Response 2.3:**

Thank you for this observation. We now explicitly clarify in the manuscript that, in the sleep/wake comparison, the external input *p* was held fixed.

Discussion:

In the sleep/wake comparison, the external input *p* was held fixed; therefore, we do not interpret ρ as the only parameter capable of reproducing these state differences, but rather as one sufficient control parameter within the parameterization explored here. For instance, in the original Jansen-Rit model, reducing the value of the input *p* can produce slow oscillation (increasing δ and θ relative power). Finally, while the model is well-suited for testing brain stimulation and drug effects in theory, these applications remain to be validated experimentally.

**Comment 2.4:**

- Few typos and missing information:

Intro: “tunning”

in 2.4 Dual fitting to EEG and fMRI -> “(define grid)” not defined….”

Etc…

**Response 2.4:**

Thank you for pointing this out. We corrected these errors in the revised manuscript, and we also fixed other minor typos and missing text throughout.

**4. REVIEWER #3**

**Comment 3.1:**
Thank you very much for this detailed and comprehensive revision. All my points have been addressed adequately, and in my view the impact and quality of the manuscript has further improved a lot. I have no further questions to add.

**Response 3.1:**
Thanks for the suggestions and your positive feedback.

**References**

1.	Lynn CW, Bassett DS. The physics of brain network structure, function and control. Nature Reviews Physics. 2019;1(5):318-32. doi: 10.1038/s42254-019-0040-8.

2.	Spiegler A, Kiebel SJ, Atay FM, Knösche TR. Bifurcation analysis of neural mass models: Impact of extrinsic inputs and dendritic time constants. NeuroImage. 2010;52(3):1041-58. doi: 10.1016/j.neuroimage.2009.12.081.

3.	Grimbert F, Faugeras O. Bifurcation Analysis of Jansen's Neural Mass Model. Neural Computation. 2006;18(12):3052-68. doi: 10.1162/neco.2006.18.12.3052.

4.	Jansen BH, Rit VG. Electroencephalogram and visual evoked potential generation in a mathematical model of coupled cortical columns. Biological Cybernetics. 1995;73(4):357-66. doi: 10.1007/BF00199471.

5.	Otero M, Lea-Carnall C, Prado P, Escobar M-J, El-Deredy W. Modelling neural entrainment and its persistence: inﬂuence of frequency of stimulation and phase at the stimulus oﬀset. Biomedical Physics & Engineering Express. 2022. doi: 10.1088/2057-1976/ac605a.

6.	Coronel‐Oliveros C, Gómez RG, Ranasinghe K, Sainz‐Ballesteros A, Legaz A, Fittipaldi S, et al. Viscous dynamics associated with hypoexcitation and structural disintegration in neurodegeneration via generative whole‐brain modeling. Alzheimer's & Dementia. 2024. doi: 10.1002/alz.13788.

7.	Stasinski J, Taher H, Meier JM, Schirner M, Perdikis D, Ritter P. Homeodynamic feedback inhibition control in whole-brain simulations. PLOS Computational Biology. 2024;20(12):e1012595.

8.	Abeysuriya RG, Hadida J, Sotiropoulos SN, Jbabdi S, Becker R, Hunt BAE, et al. A biophysical model of dynamic balancing of excitation and inhibition in fast oscillatory large-scale networks. PLOS Computational Biology. 2018;14(2):e1006007-e. doi: 10.1371/journal.pcbi.1006007.
